# Fast, heart-rate independent, whole-heart, free-breathing, three-dimensional myocardial BOLD MRI at 3T with simultaneous ^13^N-ammonia PET validation in canines

**DOI:** 10.1186/1532-429X-18-S1-W2

**Published:** 2016-01-27

**Authors:** Hsin-Jung Yang, Damini Dey, Jane M Sykes, John Butler, Behzad Sharif, Debiao Li, Sotirios Tsaftaris, Xiaoming Bi, Piotr Slomka, Frank S Prato, Rohan Dharmakumar

**Affiliations:** 1 grid.50956.3f0000000121529905Cedars Sinai Medical Center, Los Angeles, CA USA; 2 grid.415847.b0000000105562414Lawson Health Research Institute, London, ON Canada; 3 Siemens USA, Los angeles, CA USA; 4 grid.462365.00000000417909464Institute for Advanced Studies Lucca, Lucca, Italy

## Background

Myocardial BOLD MRI is a non-contrast approach for examining myocardial perfusion. Although recent developments have shown promising technical advancements, current myocardial BOLD MR methods are still limited by: (a) poor spatial coverage; (b) imaging confounders; and (c) imaging artifacts, particularly at 3T. To address these limitations, we developed a heart-rate independent, free-breathing 3D T_2_ mapping technique at 3T that utilizes near 100% imaging efficiency, which can be completed within 3 minutes. We tested our method in a canine model and validated our findings with simultaneously acquired ^13^N-ammonia PET perfusion data in a whole-body PET/MR system.

## Methods

Canines with and without LAD stenosis (n = 11) were studied in a PET/MR system. The proposed sequence was prescribed at rest and under adenosine stress (140 mg/min/kg). Dynamic ^13^N-ammonia PET scans were acquired for validation purpose. PET images were analyzed using qPET software. In healthy dogs, mean myocardial T_2_ (T2avg) were measured at rest and stress and mean myocardial blood flow (Qavg) were derived from PET images in the corresponding slices. Myocardial BOLD Response (MBOLDR = T2avg (stress):T2avg(rest)) and perfusion reserve (MPR = Qavg (stress):Qavg(rest)) were computed and compared. In the stenosis study, the affected regions were identified from the PET images and matched to the corresponding slices in BOLD data. Mean myocardial T_2_ and myocardial perfusion were measured at rest and stress in the affected and remote territories. MBOLDR and MPR from affected and remote regions were computed and compared against to each other.

## Results

**Figure 1:**
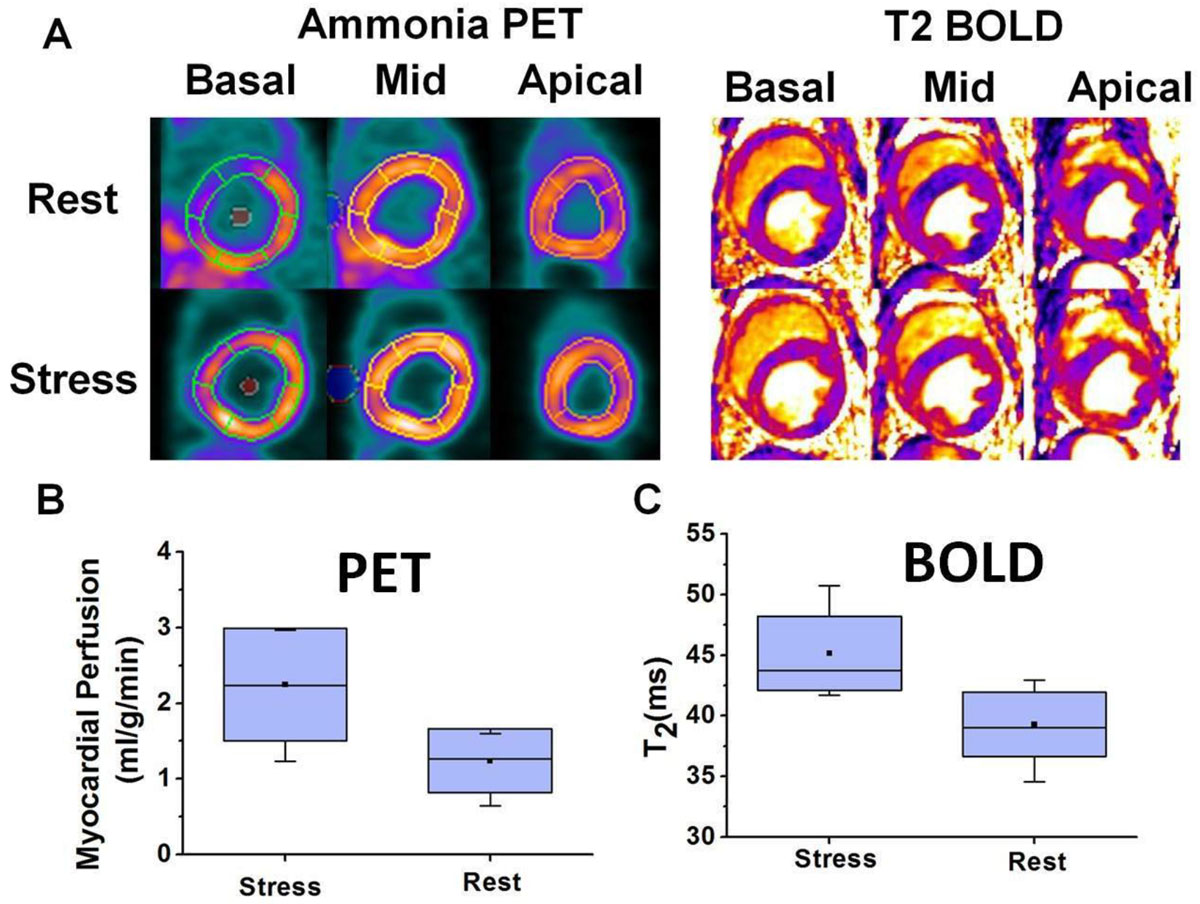
**PET MBF versus Myocardial T**_**2**_ **in Healthy Canines**. Representative short-axis PET images of myocardial blood flow and myocardial T_2_ maps at rest and stress are shown in panel A. Both BOLD and PET images demonstrate significant signal elevation during stress compared to rest. Rest and stress Mean myocardial blood flow and Myocardial T_2_ from matched slices are compared in panel B and C, respectively. An average of 2.5 fold increase of myocardial blood flow during stress is observed (panel B) along with a 15% T_2_ elevation presented in panel C.

**Figure 2:**
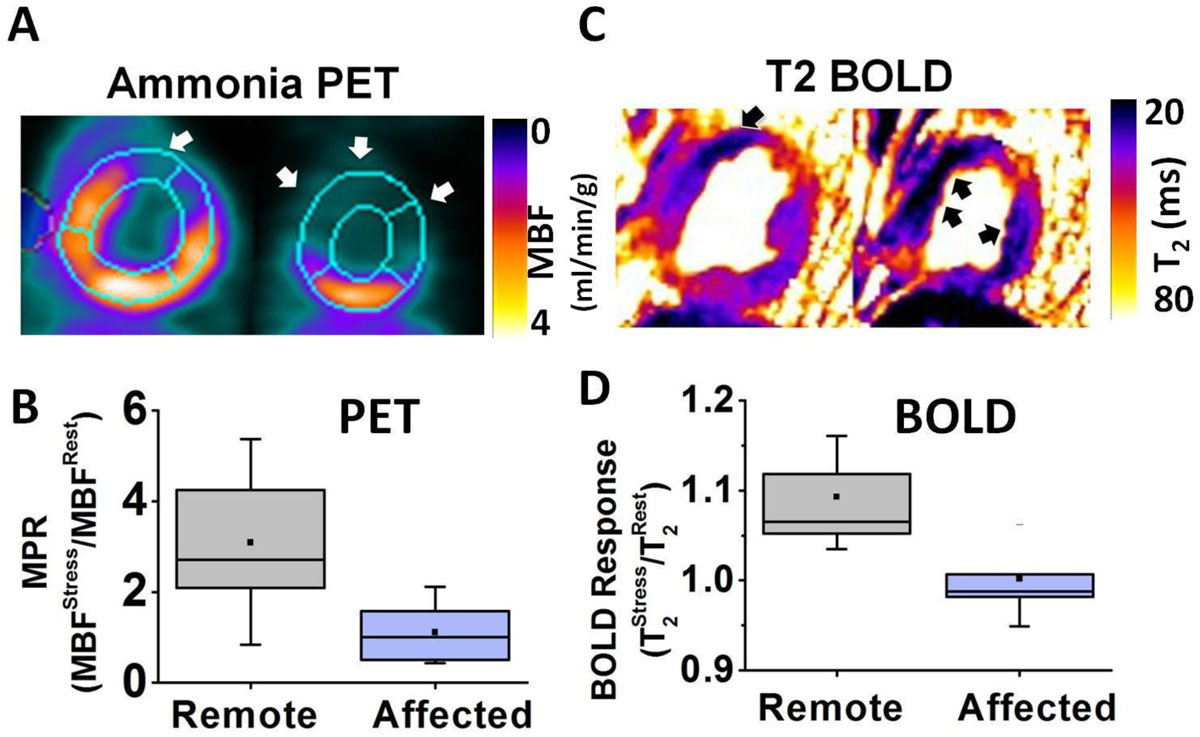
**PET MPR versus Myocardial BOLD Response in Canines with LAD Stenosis**. Representative short-axis PET images showing myocardial blood flow (panel A) and myocardial T_2_ maps (panel C) acquired during adenosine infusion are shown. Hypoperfused territories are highlighted with arrows in both PET and BOLD images. Note the close correspondence between myocardial perfusion defects identified in the PET and BOLD images. MPR and BOLD Response between remote and affected territories are compared in panels B and D, respectively. MPR in the remote territory was significantly higher than MPR in the affected territories. Similar observations were made with myocardial BOLD response (panel D).

A representative set of BOLD and PET images acquired from a healthy dog under rest and stress are shown in Figure 1. T2avg measured under stress were significantly higher than at rest (T2avg: 38.5 ± 1.0 ms (rest) vs. 44.4 ± 3.1 ms (stress), p < 0.05). As expected, Qavg were significantly higher during adenosine stress relative to rest (Qavg: 0.8 ± 0.1 ml/mg/min (rest) vs 2.0 ± 0.9 ml/mg/min (stress); p < 0.05). Linear regression of MBOLDR and MPR showed high correlation (R^2^ = 0.67, p < 0.05). In Figure 2, a set of PET and BOLD images from a dog with LAD stenosis acquired during stress are presented (A and C). Perfusion defect was consistently observed in the LAD territory from both PET and BOLD images. Panel B shows MPR was significantly higher in the remote regions (2.8 ± 1.7) compare to the affected regions (1.4 ± 1.0), p < 0.05. Significant higher MBOLDR was also observed in panel D (Remote: 1.09 ± 0.04, Affected: 1.00 ± 0.03, p < 0.05).

## Conclusions

The proposed BOLD CMR approach permits rapid whole LV assessment of BOLD changes between rest and adenosine stress. The BOLD responses were very closely correlated with PET perfusion, suggesting that the proposed BOLD CMR method is a viable approach for imaging myocardial perfusion. The method remains to be validated in patients.

